# Inferring *R*_0_ in emerging epidemics—the effect of common population structure is small

**DOI:** 10.1098/rsif.2016.0288

**Published:** 2016-08

**Authors:** Pieter Trapman, Frank Ball, Jean-Stéphane Dhersin, Viet Chi Tran, Jacco Wallinga, Tom Britton

**Affiliations:** 1 Department of Mathematics, Stockholm University, Stockholm, Sweden; 2 School of Mathematical Sciences, University of Nottingham, Nottingham, UK; 3 LAGA, CNRS (UMR 7539), Université Paris 13, Sorbonne Paris Cité, France; 4 Laboratoire Paul Painlevé, Université des Sciences et Technologies de Lille, Villeneuve-d'Ascq, France; 5 Rijksinstituut voor Volksgezondheid en Milieu (RIVM), Bilthoven, The Netherlands; 6 Department of Medical Statistics and Bioinformatics, Leiden University Medical Center, Leiden, The Netherlands

**Keywords:** infectious disease modelling, emerging epidemics, population structure, real-time spread, *R*_0_

## Abstract

When controlling an emerging outbreak of an infectious disease, it is essential to know the key epidemiological parameters, such as the basic reproduction number *R*_0_ and the control effort required to prevent a large outbreak. These parameters are estimated from the observed incidence of new cases and information about the infectious contact structures of the population in which the disease spreads. However, the relevant infectious contact structures for new, emerging infections are often unknown or hard to obtain. Here, we show that, for many common true underlying heterogeneous contact structures, the simplification to neglect such structures and instead assume that all contacts are made homogeneously in the whole population results in conservative estimates for *R*_0_ and the required control effort. This means that robust control policies can be planned during the early stages of an outbreak, using such conservative estimates of the required control effort.

## 1. Introduction

An important area of infectious disease epidemiology is concerned with the planning for mitigation and control of new emerging epidemics. The importance of such planning has been highlighted during epidemics over recent decades, such as human immunodeficiency virus (HIV) around 1980 [1], severe acute respiratory syndrome (SARS) in 2002/2003 [2], the influenza A H1N1 pandemic in 2009 [3] and the Ebola outbreak in West Africa, which started in 2014 [4]. A key priority is the early and rapid assessment of the transmission potential of the emerging infection. This transmission potential is often summarized by the expected number of new infections caused by a typical infected individual during the early phase of the outbreak, and is usually denoted by the basic reproduction number, *R*_0_. Another key priority is estimation of the proportion of infected individuals we should isolate before they become infectious, and thus completely prevent them from spreading the disease to any other individuals in order to break the chain of transmission. This quantity is denoted as the required control effort, *v*_c_. From a modelling perspective, *v*_c_ is equivalent to the critical vaccination coverage: if a vaccine is available, then the required control effort is equal to the proportion of the population that needs to be immunized in order to stop the outbreak, if the immunized people are chosen uniformly at random. These key quantities are inferred from available observations on symptom onset dates of cases and the generation times, i.e. the typical duration between time of infection of a case and infection of its infector [5,6]. The inference procedure for *R*_0_ and *v*_c_ requires information on the infectious contact structure (‘who contacts whom’), information that is typically not available or hard to obtain quickly for emerging infections.

The novelty of this paper lies in that we assess the estimators for the basic reproduction number *R*_0_ and required control effort *v*_c_, which are based on usually available observations, over a wide range of assumptions about the underlying infectious contact structure. We find that most plausible contact structures result in only slightly different estimates of *R*_0_ and *v*_c_. Furthermore, we find that ignoring the infectious contact pattern, thus effectively assuming that individuals mix homogeneously, will in many cases result in a slight *overestimation* of these key epidemiological quantities, even if the actual contact structure is far from homogeneous. This is important good news for planning for mitigation and control of emerging infections, because the relevant contact structure is typically unknown: ignoring the contact structures results in slightly conservative estimates for *R*_0_ and *v*_c_. This is a significant justification for basing infection control policies on estimates of *R*_0_ derived for the Ebola outbreak in West Africa in [4], for which, although we know that transmission is mainly due to close and intimate contact with bodily fluids, it is hard to obtain data on who regularly has such contact with whom. Therefore, the data are stratified by region, without further assumptions on contact structure.

We focus on communicable diseases in a closed population (i.e. a population without births, migration and non-disease-related deaths) that follow an infection cycle where the end of the infectious period is followed by long-lasting immunity or death. In such an infection cycle, individuals are either susceptible, exposed (latently infected), infectious or removed, which means either recovered and permanently immune (or immune for the duration of the epidemic) or dead. Those dynamics can be described by the so-called stochastic SEIR epidemic model [7, ch. 3]. For ease of presentation, we use the Markov SIR epidemic as a leading example. In this special case, there is no latent period (so an individual is able to infect other individuals as soon as they are infected), the infectious period is exponentially distributed with expected length 1/*γ*, and infected individuals make close contacts at a constant rate *λ*. While infectious, an individual infects all susceptible individuals with whom he or she has close contact. The rate at which an infectious individual makes contact with other individuals depends on the contact structure in the community but it does not change over time in the Markov SIR model. The more general results for the full SEIR epidemic model are given and derived in the electronic supplementary material.

**Figure 1.**
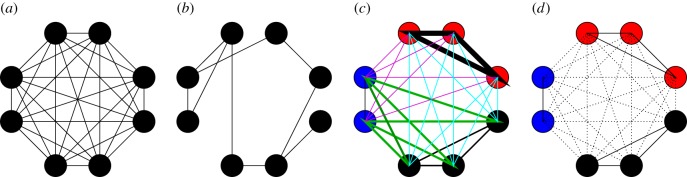
The four contact structures considered: individuals are represented by circles and possible contacts are denoted by lines between them. (*a*) A homogeneously mixing population, in which all individuals have the same frequency of contacting each other. (*b*) A network-structured population, in which, if contact between two individuals is possible, the contacts occur at the same frequency. (*c*) A multi-type structure with three types of individuals, in which individuals of the same type have the same colour and lines of different colour and width represent different contact frequencies. (*d*) A population partitioned into three households, in which members of the same households have the same colour and household contacts, represented by solid lines, have higher frequency than global contacts, represented by dotted lines.

We cover a wide range of possible contact structures. For each of these, we derive estimators of the basic reproduction number and the required control effort. We start with the absence of structure, when the individuals mix homogeneously [8, ch. 1] (figure 1*a*). We examine three different kinds of heterogeneities in contacts: the first kind, network structure [9–12] (figure 1*b*), emphasizes that individuals have regular contacts with only a limited number of other individuals; the second kind, multi-type structure (figure 1*c*), emphasizes that individuals can be categorized into different types, such as age classes, where differences in contact behaviour with respect to disease transmission are pronounced among individuals of different type but negligible among individuals of the same type [7,13]; and the third kind, household structure [14,15] (figure 1*d*), emphasizes that individuals tend to make most contacts in small social circles, such as households, school classes or workplaces. Finally, we compare the performance of the estimators for *R*_0_ and *v*_c_ against the simulated spread of an epidemic on an empirical contact network.

## 2. Estimation of *R*_0_ and required control efforts for various contact structures

### 2.1. Homogeneous mixing

Many results for epidemics in large homogeneously mixing populations can be obtained, because the initial phase of the epidemic is well approximated by a branching process [16–18], for which an extensive body of theory is available. In particular, an outbreak can become large only if *R*_0_ > 1. Note that if *R*_0_ > 1, then it is still possible that the epidemic will go extinct quickly. The probability for this to happen can be computed [7, eqn 3.10] and is less than 1. Another result is that if *R*_0_ > 1 and the epidemic grows large (which we assume from now on), then the number of infectious individuals grows roughly proportional to *e^αt^* during the initial phase of the epidemic. Here, *t* is the time since the start of the epidemic and the epidemic growth rate *α* is a positive constant, which depends on the parameters of the model, through the equation2.1
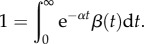
Here, *β*(*t*) is the expected rate at which an infected individual infects other individuals *t* time units after they were infected. For the Markov SIR model, with expected duration of the infectious period 1/γ, *β*(*t*) is given by 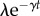. This can be understood by observing that *λ* is the rate at which an infected individual makes contacts if he or she is still infectious, whereas 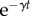 is the probability that the individual is still infectious *t* time units after he or she became infected. The epidemic growth rate *α* corresponds to the Malthusian parameter for population growth. Note that the expected number of newly infected individuals caused by a given infected individual is2.2
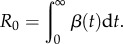
For the Markov SIR model, (2.1) and (2.2) translate to2.3
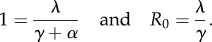
Because we usually have observations on symptom-onset dates of cases for a new, emerging epidemic, as was the case for the Ebola epidemic in West Africa, it is often possible to estimate *α* from observations. In addition, we often have observations (albeit often only for a subset of the infected cases) on the typical duration between time of infection of a case and infection of its infector, which allow us to estimate, assuming a Markov SIR model, the average duration of the infectious period, 1/*γ* [5]. Using (2.3), this provides us with an estimator of *R*_0_ in a homogeneously mixing Markov SIR model2.4
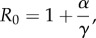
which, as desired, does not depend on *λ*. In the electronic supplementary material, we deduce expressions for *α* and *R*_0_, in terms of the model parameters for the more general SEIR epidemic, and relate these quantities.

The required control effort for the SEIR epidemic in a homogeneously mixing population is known to depend solely on *R*_0_ through the relation [7, p. 69]2.5
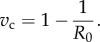
Thus, we obtain an estimator of the required control effort in terms of observable growth rate and duration of the infectious period2.6
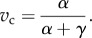
We compare the estimators (2.4) and (2.6) with other estimators that we obtain for different infectious contact structures, using the same values for the epidemic growth rate and duration of the infectious period. Throughout the comparison, we assume that the initial stage of an epidemic shows exponential growth, which is a reasonable assumption for many diseases, including the Ebola epidemic in West Africa.

### 2.2. Network structure

One kind of infectious contact structure is network structure. We consider the so-called configuration model ([19], [20, ch. 3]) in which each individual may contact only a limited number (which varies between individuals) of other acquaintances, with mean *μ* and variance *σ*^2^. In such a network, the mean number of different individuals (acquaintances) a typical newly infected individual can contact (other than his or her infector) is referred to as the mean excess degree [19], which is given by
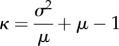
(see the electronic supplementary material or [19] for the derivation of *κ*). This quantity is hard to observe for a new emerging infection, but we know the value must be finite and strictly greater than 1 if the epidemic grows exponentially fast. For the Markov SIR model for which the constant rate at which close contacts per pair of acquaintances occur is denoted by *λ*^(net)^, we obtain 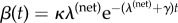. This can be seen by noting that *κ* is the expected number of susceptible acquaintances a typical newly infected individual has in the early stages of the epidemic, whereas 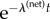 is the probability that a given susceptible individual is not contacted by the infective over a period of *t* time units, and 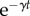 is the probability that the infectious individual is still infectious *t* time units after he or she became infected. In the electronic supplementary material, we deduce an estimator of *R*_0_ in terms of the observable epidemic growth rate, the average duration of the infectious period and the unobservable mean excess degree: 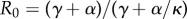 (cf. [21]). We find that the estimator obtained assuming homogeneous mixing (2.4) overestimates *R*_0_ by a factor 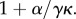


We know that this factor is strictly greater than 1, because the exponential growth rate *α*, the recovery rate *γ* and the mean excess degree *κ* (which is often hard to observe) are all strictly positive. Furthermore, the factor tends to 1 as *κ* tends to infinity.

In the electronic supplementary material, we also consider more general SEIR models. We conclude that estimates of *R*_0_ obtained by assuming homogeneous mixing are always larger than the corresponding estimates if the contact structure follows the configuration network model. In the electronic supplementary material, we also show, by example, that if we allow for even more general random infection cycle profiles, then it is possible that assuming homogeneous mixing might lead to a non-conservative estimate of *R*_0_. However, for virtually all standard models studied in the literature, assuming homogeneous mixing leads to conservative estimates.

As is the case for the homogeneously mixing contact structure, the required control effort for epidemics on the network structures under consideration is known to depend solely on *R*_0_ through equation (2.5) [22]. This provides us with an estimator of *v*_c_ in terms of observable *α* and duration of infectious period and the unobservable mean excess degree *κ*: 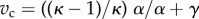. We find that the estimator obtained assuming homogeneous mixing overestimates *v*_c_ by a factor 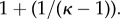 This factor is always strictly greater than 1, because the mean excess degree *κ* is strictly greater than 1, and again tends to 1 as *κ* tends to infinity. Thus, *v*_c_ obtained by assuming homogeneous mixing is always larger than that of the configuration network model. Consequently, we conclude that, if the actual infectious contact structure is made up of a configuration network and a perfect vaccine is available, we need to vaccinate a smaller proportion of the population than predicted assuming homogeneous mixing.

**Figure 2.**
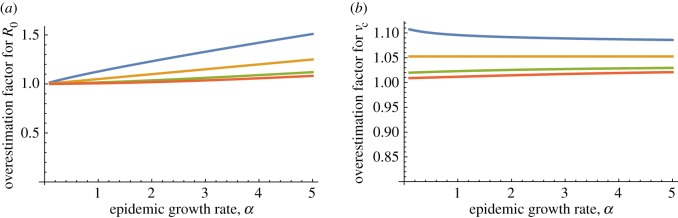
The factor by which estimators based on homogeneous mixing will overestimate (*a*) the basic reproduction number *R*_0_ and (*b*) the required control effort *v*_c_ for the network case. Here, the epidemic growth rate *α* is measured in multiples of the mean infectious period 1/*γ*. The mean excess degree *κ* = 20. The infectious periods are assumed to follow a gamma distribution with mean 1 and standard deviation *σ* = 1.5, *σ* = 1, *σ* = 1/2 and *σ* = 0, as displayed from top to bottom. Note that the estimate of *R*_0_ based on homogeneous mixing is 1 + *α*. Furthermore, note that *σ* = 1 corresponds to the special case of an exponentially distributed infectious period, whereas if *σ* = 0 the duration of the infectious period is not random. (Online version in colour.)

The overestimation of *R*_0_ is small whenever *R*_0_ is not much larger than 1 or when *κ* is large. The same conclusion applies to the required control effort *v*_c_. The observation that the *R*_0_ and *v*_c_ for the homogeneously mixing model exceed the corresponding values for the network model extends to the full epidemic model allowing for an arbitrarily distributed latent period followed by an arbitrarily distributed independent infectious period, during which the infectivity profile (the rate of close contacts) may vary over time but depends only on the time since the start of the infectious period (see the electronic supplementary material for the corresponding equations). Figure 2*a* shows that, for SIR epidemics with gamma-distributed infectious periods, the factor by which the homogeneous mixing estimator overestimates the actual *R*_0_ increases with increasing epidemic growth rate *α*, and suggests that this factor increases with increasing standard deviation of the infectious period. Figure 2*b* shows that the factor by which the homogeneous mixing estimator overestimates the actual *v*_c_ decreases with increasing *α* and increases with increasing standard deviation of the infectious period. When the standard deviation of the infectious period is low, which is a realistic assumption for most emerging infectious diseases [23], and *R*_0_ is not much larger than 1, then ignoring the contact structure in the network model and using the simpler estimates based on homogeneous mixing results in a slight overestimation of *R*_0_ and *v*_c_.

### 2.3. Multi-type structure

A second kind of infectious contact structure reflects that often a community contains different types of individuals that display specific roles in contact behaviour. Types might be related to age groups, social behaviour or occupation. It may be hard to classify all individuals into types and sometimes data on the types of individuals are missing. Furthermore, the number of parameters required to describe the contact rates between the types is large. We assume that there are *K* types of individuals, labelled 1,2, … ,*K*, and that for *i* = 1, … ,*K* a fraction *π_i_* of the *n* individuals in the population is of type *i*. For the Markov SIR epidemic, we assume that the rate of close contacts from a given type *i* individual to a given type *j* individual is *λ_ij_*/*n*. Note that here close contacts are not necessarily symmetric, i.e. if individual *x* makes a close contact with individual *y*, then it is not necessarily the case that *y* makes a close contact with *x*. We assume again that individuals stay infected for an exponentially distributed time with expectation 1/*γ*. The expected rate at which a given type *i* individual infects type *j* individuals at time *t* since infection is 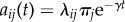. Here, *λ_ij_*/*n* is the rate at which the type *i* individual contacts a given type *j* individual, *nπ_j_* is the number of type *j* individuals and 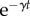 is the probability that the type *i* individual is still infectious *t* time units after being infected. It is well known [7,13,24,25] that the basic reproduction number 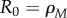 is the largest eigenvalue of the matrix *M*, which has elements 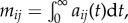 and the epidemic growth rate *α* is such that 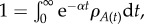 where 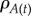 is the largest eigenvalue of the matrix *A*(*t*) with elements 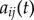. Let *ρ* be the largest eigenvalue of the matrix with elements 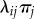 and note that 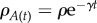. Therefore,
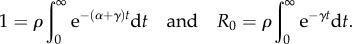
These equalities imply that
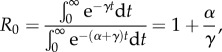
which shows that the relation between *R*_0_ and *α* for this class of multi-type Markov SIR epidemics is the same as for such an epidemic in a homogeneously mixing population (cf. equation (2.4)).

It is readily seen that if for every type of individual a fraction 1 − *v*_c_ is immunized, then the expected number of individuals infected by one infectious individual decreases by a factor 1 − *v*_c_, for all types of individuals. This implies that, for epidemics in a multi-type population structure, the relation 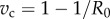 still holds. In the electronic supplementary material, we derive that estimators for *R*_0_ and (if control measures are independent of the types of individuals) *v*_c_ are *exactly* the same as for homogeneous mixing in a broad class of SEIR epidemic models. This class includes the full epidemic model allowing for arbitrarily distributed latent and infectious periods and models in which the rates of contacts between different types keep the same proportion all of the time, although the rates themselves may vary over time (cf. [24]).

We illustrate our findings on multi-type structures through simulations of SEIR epidemics in §3.1.

### 2.4. Household structure

**Figure 3.**
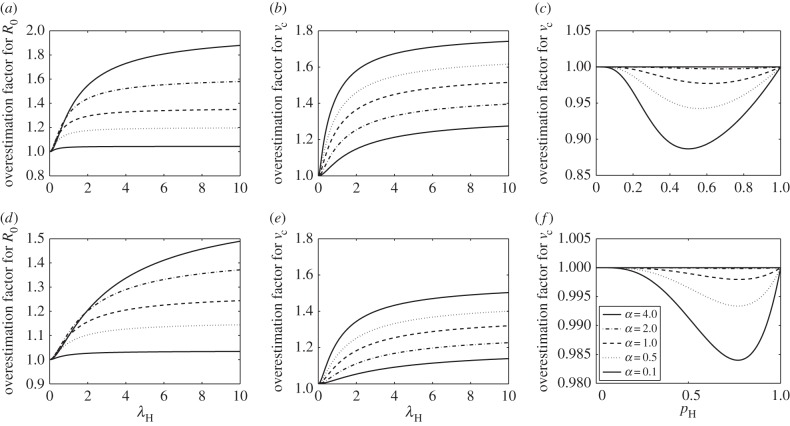
The factor by which estimators based on homogeneous mixing overestimate key epidemiological variables in a population structured by households. The basic reproduction number *R*_0_ for Markov SIR epidemics with expected infectious period equal to 1 (*a,d*), critical vaccination coverage *v*_c_ for Markov SIR epidemics (*b,e*) and *v*_c_ for Reed–Frost epidemics (*c,f*), as a function of the relative influence of within-household transmission, in a population partitioned into households. For (*a*–*c*), the household size distribution is taken from a 2003 health survey in Nigeria [29] and is given by 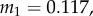 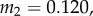 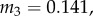 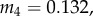 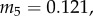 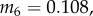 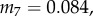 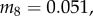 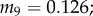 for 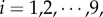 *m_i_* is the fraction of the households with size *i*. For (*d*–*f*), the Swedish household size distribution in 2013 taken from [30] is used and is given by 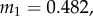 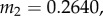 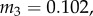 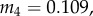 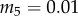. The global infectivity is chosen, so that the epidemic growth rate *α* is kept constant while the within-household transmission varies. Homogeneous mixing corresponds to 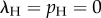, in which case 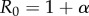. Note that the order of the graphs is different in (*b*) and (*e*) from that in (*a*,*c*,*d*,*f*).

A third kind of infectious contact structure is household structure. This partitions a population into many relatively small social groups or households, which reflect actual households, school classes or workplaces. This contact structure is different from the multi-type structure, because, in the latter, the population is partitioned into a limited number of large groups of individuals having the same type. The contact rate between pairs of individuals from different households is small and the contact rate between pairs of individuals in the same household is much larger. This model was first analysed in detail in [15]. It is possible to define several different measures for the reproduction numbers for this model [14,26], but the best suited for our purpose is given in [27,28]. For this model, it is hard to find explicit expressions for *R*_0_ and required control effort in terms of the observable epidemic growth rate. Numerical computations described in [28] suggest that the difference between the estimated *R*_0_ based on *α* and the real *R*_0_ might be considerable, but it is theoretically shown that the estimate is conservative for the most commonly studied models. It is also argued that the required control effort 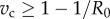 (with equality if and only if all households have size less than or equal to 3) for this model, which implies that, if we know *R*_0_ and we base our control effort on this knowledge, we might fail to stop an outbreak. However, we usually do not have direct estimates for *R*_0_, and even though it is not true in general that using *R*_0_ leads to conservative estimates for *v*_c_ [28] numerical computations suggest that the approximation of *v*_c_ using *α* and the homogeneous mixing assumption is often conservative. This is illustrated in figure 3, which shows the factors by which the homogeneous mixing estimators overestimate the true *R*_0_s and *v*_c_s over a range of values for the relative contribution of the within-household spread. We use two types of epidemics: in (*a*) and (*b*), the Markov SIR epidemic is used, whereas in (*c*), the so-called Reed–Frost model is used, which can be interpreted as an epidemic in which infectious individuals have a long latent period of non-random length, after which they are infectious for a very short period of time. We note that for the Reed–Frost model the relationship between *α* and *R*_0_ does not depend on the household structure (cf. [28]) and therefore, for this model, only the dependence of *v*_c_ on the relative contribution of the within-household spread is shown in figure 3. The household size distributions are taken from a 2003 health survey in Nigeria [29] and from data on the Swedish household size distribution in [30]. For Markov SIR epidemics, as the within-household infection rate *λ*_H_ is varied, the global infection rate is varied in such a way that the computed epidemic growth rate *α* is kept fixed. For this model, *α* is calculated using the matrix method described in §4.1 of [31]. In figure 3*a*,*b*,*d,e,* we observe that the overestimation factor for *R*_0_ increases with *α,* whereas that for *v*_c_ decreases with *α*. For the Reed–Frost epidemic model, the probability that an infectious individual infects a given susceptible household member during its infectious period, *p*_H_, is varied, whereas the corresponding probability for individuals in the general population varies with *p*_H_, so that *α* is kept constant. For this model, assuming that the unit of time is the length of the latent period, *R*_0_ coincides with the initial geometrical rate of growth of infection, so *α* = log(*R*_0_). From figure 3, we see that estimates of *v*_c_ assuming homogeneous mixing are reliable for Reed–Frost-type epidemics, although, as opposed to all other analysed models and structures, the estimates are not conservative. We see also that, for the Markov SIR epidemic, estimating *R*_0_ and *v*_c_ based on the homogeneous mixing assumption might lead to conservative estimates which are up to 80% higher than the real *R*_0_ and *v*_c_.

The results obtained for Markov SIR epidemics in the homogeneously mixing, network and multi-type population structures are summarized in table 1. The results from household models are not in the table, because determining *α*, *R*_0_ and *v*_c_ requires solutions of nonlinear equations, which themselves are rather complex and defined only recursively.

**Table 1.** The epidemic growth rate *α*, the basic reproduction number *R*_0_ and required control effort *v*_c_ for a Markov SIR epidemic model as functions of the model parameters in the homogeneously mixing, network and multi-type models and their relationships to each other.

model	quantity of interest	quantity of interest as function of		ratio with homogeneous mixing
		*λ*, *γ* and *κ*	*α*, *γ* and *κ*	
homogeneous mixing	*α*	*λ* − *γ*	—	—
	*R*_0_	*λ*/*γ*	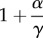	—
	*v*_c_	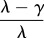	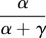	—
network	*α*	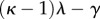	—	—
	*R*_0_	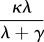	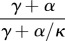	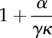
	*v*_c_	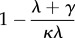	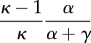	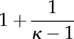
multi-type	*α*	*γ*(*ρ_M_* − 1)	—	—
	*R*_0_	*ρ_M_*	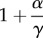	1
	*v*_c_	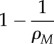	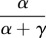	1

## 3. Simulation studies

### 3.1. Simulation of an epidemic in a multi-type population structure

**Figure 4.**
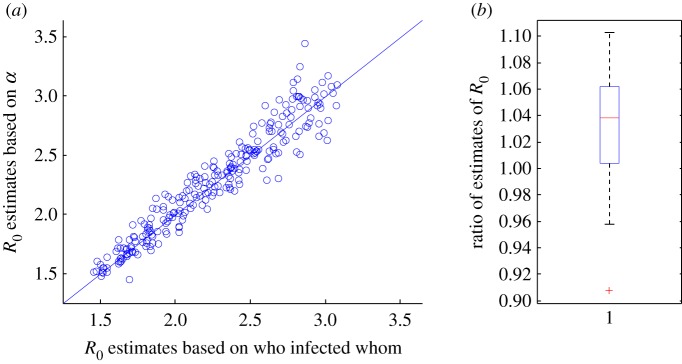
The estimated basic reproduction number, *R*_0_, for a Markov SEIR model in a multi-type population as described in [32], based on the real infection process (who infected whom) plotted against the computed *R*_0_, assuming homogeneous mixing, based on the estimated epidemic growth rate, *α*, and given expected infectious period (5 days) and expected latent period (10 days). The infectivity is chosen at random, such that the theoretical *R*_0_ is uniform between 1.5 and 3. The estimate of *α* is based on the times when individuals become infectious. In (*b*), a box plot of the ratios of the two *R*_0_ estimates (the estimate based on the homogeneous mixing assumption divided by the estimate based on the real infection process for each of the 250 simulation runs) is given. (Online version in colour.)

We illustrate our findings on multi-type structures through simulations of SEIR epidemics in an age-stratified population with known contact structure. As a population, we took the Dutch population in 1987 (approx. 14.6 million people) as used in [32], for which extensive data on contact structure are available. The population is subdivided into six age groups, and contact intensities are based on questionnaire data. Further details on the population, their types and contact intensities can be found in the electronic supplementary material. We use values of the average infectious period 1/*γ* and the average latent period 1/*δ* close to the estimates for the 2014 Ebola epidemic in West Africa [4]. The simulation and estimation methods are described in detail in the electronic supplementary material. We use two estimators for *R*_0_. The first of these estimators is based on the average number of infections among the people who were infected early in the epidemic. This procedure leads to a good estimate of *R*_0_ if the spread of the disease is observed completely. The second estimator for *R*_0_ is based on 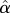, an estimate of the epidemic growth rate *α*, and known expected infectious period 1/*γ* and expected latent period 1/*δ*, and is given by 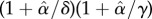. We calculate estimates of *R*_0_ using these two estimators for 250 simulation runs. As predicted by the theory, the simulation results show that for each run the estimates are close to the actual value without a systematic bias (figure 4 and electronic supplementary material, figure S1). Note that in figure 4 we compare two estimators of *R*_0_, which are each based on a finite number of observations and hence not exact. We do not compare the estimates of *R*_0_ with the computed value of *R*_0_ based on the model parameters.

### 3.2. Estimation of *R*_0_ and required control efforts for empirical network structure

The three kinds of infectious contact structure studied are caricatures of actual social structures. Those actual structures may contain features of all three caricatures, and reflect small social groups such as school classes and households in which individuals interact frequently, as well as distinct social roles such as those based on age and gender, and frequently repeated contacts among those acquaintances. This leads us to expect that estimators based on ignoring contact structure will in general result in a slight overestimation of *R*_0_ and required control effort.

**Figure 5.**
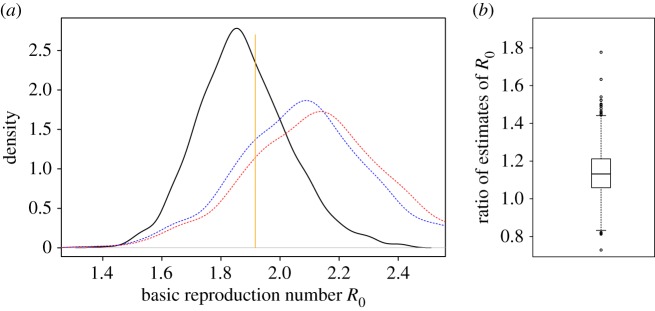
Estimates for the basic reproduction number *R*_0_ of an SEIR epidemic on the collaboration network in condensed matter physics [33] based on 1000 simulated outbreaks. Each epidemic is started by 10 individuals chosen uniformly at random from the 23 133 individuals in the population. The infection rate is chosen such that 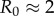. In (*a*), the black solid line provides the density of estimates based on full observation of who infected whom, the blue dashed line denotes the density of estimates based on the estimated epidemic growth rate *α* and the assumption that the network is a configuration model with known *κ*, whereas the red dotted line denotes the density of estimates based on *α* and the homogeneous mixing assumption. (The modes of these three densities are in increasing order.) The orange vertical line segment denotes the estimate of *R*_0_ based only on the infection parameters and *κ*, assuming that the network is a configuration model (see equation (2.12) in the electronic supplementary material). We excluded the 50 simulations with highest estimated *α* and the 50 simulations with lowest estimated *α*. In (*b*), a box plot of the ratios of the two *R*_0_ estimates (the estimate based on the homogeneous mixing assumption divided by the estimate based on the real infection process for each of the 250 simulation runs) is provided. (Online version in colour.)

We test this hypothesis further on some empirical networks taken from the Stanford Large Network Dataset Collection [33]. In this report, we present a network of collaborations in condensed matter physics, where the individuals are authors of papers and authors are ‘acquaintances’ if they were co-authors of a paper posted on the e-print service arXiv in the condensed matter physics section between January 1993 and April 2004. In the electronic supplementary material, we also analyse SEIR epidemics on two other networks from [33]. The ‘condensed matter physics’ network is built up of many (overlapping) groups that represent papers. It was chosen since it is relatively large (23 133 individuals and 93 497 links), with over 92% of the individuals in the largest component. The mean excess degree, *κ*, for this network is approximately 21 and small groups in which everybody is acquainted with everybody else are also present. In figure 5, we show the densities of estimates of *R*_0_, based on 1000 simulations of an SEIR epidemic on this network, using parameters close to estimates for the spread of Ebola virus in West Africa [4]. The estimates are based on who infected whom in the real infection process (black line), the estimated epidemic growth rate and the configuration network assumption with 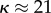 (blue dashed line) and the estimated epidemic growth rate and the homogeneous mixing assumption (red dotted line). In most of the cases (886 out of 1000), the estimate of *R*_0_ based on homogeneous mixing is larger than the estimate based on who infected whom. In only 21 out of 1000 cases, the estimate of *R*_0_ based on homogeneous mixing is less than 90% of the estimate of *R*_0_ based on who infected whom. Half of the estimates of *R*_0_ based on the epidemic growth rate and the homogeneous mixing assumption are between 12% and 45% larger than the estimate based on who infected whom. The difference in estimates might be explained through the relatively small average number of acquaintances per individual and the structure of small groups in which all individuals are acquaintances with all other individuals in the group. As in figure 4, we note that in figure 5 we compare two estimators of *R*_0_. It is hard, if not impossible, to define, let alone compute, *R*_0_ for epidemics on empirical networks.

In order to check the sensitivity of our results to the parameter values, we also performed simulations on the ‘condensed matter physics’ network with two alternative infection rates (70% and 200% of the value used in the main simulation). The qualitative results are the same as for the original simulations. We note, however, that if the infection rate is increasing the overestimation factor also shows an increasing trend. This is consistent with our observations in §§2.2 and 2.4.

## 4. Discussion and conclusion

In calculating the required control effort *v*_c_, we have assumed that vaccinations, or other interventions against the spread of the emerging infection, are distributed uniformly at random in the population. For new, emerging infections, this makes sense when we have little idea about the contact structure, and we do not know who is at high risk and who is at low risk of infection. When considering control measures that are targeted at specific subgroups, such as vaccination of the individuals at highest risk, closure of schools or travel restrictions, more information on infectious contact structure becomes essential to determine which intervention strategies are best. We note that for non-targeted control strategies the overestimation of *R*_0_ seems to be less for network-structured and multi-type populations than for populations structured in households, especially for high values of *R*_0_. Because, for epidemics among households, better strategies than non-targeted control efforts are available [15,34,35], household (and workplace) structure is the first contact structure that should be taken into account.

Overestimation of the required control effort leads to additional costs, both monetary and societal. These costs can be viewed as the value of information on the detailed contact structure of the population, because they would have been avoided had the correct details on the contact structure been incorporated into the epidemic model. This implies that obtaining the detailed contact structure could become a relevant policy option when the additional costs for infection control are sufficiently high. However, an important concern for most policy-makers is the cost of getting the decision wrong. This would require good estimates on the probabilities of extreme values of *R*_0_ (given the observed data). Even though this is clearly beyond the scope of this study, obtaining more information on such extreme values is a worthwhile objective for future work.

When the objective is to assess *R*_0_ and *v*_c_ from the observed epidemic growth rate of a new emerging infectious disease such as Ebola, ignoring contact structure leads to a positive bias in the estimated value. For both SIR epidemics and SEIR epidemics (see electronic supplementary material), this bias is small when the standard deviation of the infectious period is small enough compared with the mean, as is the case for the Markov SEIR epidemic and even more so for the Reed–Frost model. For Ebola in West Africa, we know that the standard deviation of the time between onset of symptoms (which is a good indication of the start of the infectious period) and the time until hospitalization or death is of the same order as the mean. The same holds for the time between infection and onset of symptoms [4]. These ratios of mean and standard deviation are well captured by the Markov SEIR epidemic.

Our findings are important for prioritizing data collection during an emerging epidemic, when assessing the control effort is a priority: it is most crucial to obtain accurate estimates for the epidemic growth rate from times of symptom onset of cases, and duration of the infectious and latent periods from data on who acquires infection from whom [36–38]. This is consistent with current practice [4,39]. Data about the contact structure will be welcome to add precision, but will have little effect on the estimated non-targeted required control effort in an emerging epidemic.

Throughout the manuscript, we assume that we have enough data for reliable estimates of *α*. Further research on evaluating the behaviour of the estimators themselves in finite structured populations is needed, but beyond the scope of the present research.

## Supplementary Material

Supporting information for: Inferring R0 in emerging epidemics, the effect of common population structure is small

## Data accessibility

No primary data are used in this article. Secondary data sources are taken from the Stanford Large Network Dataset Collection [33], from [29,30] and from [32].
