# Supplementary material for: Inferring R0 in emerging epidemics—the effect of common population structure is small
Source: J R Soc Interface. 2016 Aug;13(121):20160288. doi: 10.1098/rsif.2016.0288 (PMC5014060; doi:10.1098/rsif.2016.0288)
Supplement: Supporting information for: Inferring R0 in emerging epidemics, the effect of common population structure is small [file rsif20160288supp1.pdf]

Supporting information for: Inferring R_0 in emerging epidemics – the effect of common population structure is small

Pieter Trapman,[1*] Frank Ball,[2] Jean-Stéphane Dhersin,[3]
Viet Chi Tran,[4] Jacco Wallinga,[5,6] and Tom Britton[1]

[1]Department of Mathematics, Stockholm University, Sweden
[2]School of Mathematical Sciences, University of Nottingham, UK
[3]LAGA, CNRS (UMR 7539), Université Paris 13, Sorbonne Paris Cité, France
[4]Laboratoire Paul Painlevé, Université des Sciences et Technologies de Lille, France
[5]Rijksinstituut voor Volksgezondheid en Milieu (RIVM), Bilthoven, The Netherlands
[6]Department of Medical Statistics and Bioinformatics,
Leiden University Medical Center, Leiden, The Netherlands

In this supplementary material we discuss the mathematics behind some of the claims in the main article, how simulations are performed and how estimates are obtained from the simulations. The parameters used for the Markov SEIR epidemic model are summarized in Table 1.

1 Mathematical methods

1.1 Introduction

The stochastic and mathematical analysis of the spread of infectious diseases in large populations often relies on the theory of branching processes [1]. Branching processes are introduced as a model to describe family trees, where the simplifying assumption is that all women (in the branching process literature often the female lines are chosen) have the same probability, p_k, of having k daughters, where k can be any non-negative integer. Furthermore, the numbers of daughters of different women are independent.

It is clear that this model ignores important properties of real populations, such as changing circumstances which make the distribution of the number of children change over time and the fact that populations in general cannot grow indefinitely because of competition for resources. However, simple as it is, the model has proved useful in many situations.

Branching processes are also useful to describe the spread of SEIR (susceptible → exposed → infectious → recovered/removed) epidemics, where an infection can be seen as a birth, with the infector being the mother and the infectee the daughter. In this model

general parameters and notation	
λ	infection rate
$1/\gamma$	average duration of infectious period
$1/\delta$	average duration of latent period
α	exponential growth rate of number of infected individuals
n	population size
R_0	basic reproduction number, transmission potential, mean number of new infections caused by typical infected individual
v_c	required control effort, critical vaccination coverage
$I(t)$	number of infectious individuals at time t
parameters specific for network model	
μ	average number of acquaintances of individuals
σ^2	variance of the number of acquaintances
κ	the mean number of acquaintances of newly infected individual, excluding the infector, $\kappa = \frac{\sigma^2}{\mu} + \mu - 1$
parameters specific for multi-type model	
ι	number of different types
π_j	fraction of population with type j
λ_{ij}	infection rate from type i to type j individual
M	$\iota \times \iota$ next generation matrix, with elements $m_{ij} = \lambda_{ij}\pi_j/\gamma$
J	$\iota \times \iota$ identity matrix
ρ_A	largest eigenvalue of matrix A

Table 1: Parameters and notation used for SEIR epidemic model in homogeneously mixing populations, on networks and in multi-type populations

competition for resources is apparent, since once a susceptible individual is infected it cannot be infected again. However, if the population size n is large and the number of no-longer-susceptible individuals is of smaller order than $\sqrt{n}$, then in homogeneous mixing populations, in configuration model network populations, in household models and in multi-type population models, suitable branching process approximations are very good (see e.g. [2]) and we use them without further justification. In the following paragraphs and sections, we discuss branching processes using terminology borrowed from epidemics.

Branching processes can be analysed in real time and in generations. In real time, the Malthusian parameter or the epidemic growth rate, α is arguably the most important parameter. A key theorem in branching processes [1, Thm.6.8.1] states that if the number of infected individuals in the population grows large, then it roughly grows at a rate proportional to $e^{\alpha t}$, where t is the time since the infectious disease was introduced. From a generation perspective the essential parameter is R_0, the basic reproduction number, i.e. the average number of infections per typical infectious individual in an otherwise fully susceptible population. An outbreak can become large only if $R_0 > 1$, which happens if and only if $\alpha > 0$. Note that if $R_0 > 1$, then it is still possible that the epidemic will go extinct quickly. The probability for this to happen can be computed [3, Eq. 3.10] and is less than 1.

In the remainder of this supplementary material, we first discuss some useful results from the theory of branching processes. Then we apply them to epidemics in respectively homogeneously mixing populations, network populations, multi-type populations and household populations. Throughout we focus on R_0. It is however worth remarking that in homogeneously mixing populations, in (configuration model) network populations and in multi-type populations, we can deduce straightforwardly the required control effort or critical vaccination coverage, v_c from R_0 (see main text). For more extensive discussions on control effort and vaccination in the household model see [4]. We note that the critical vaccination coverage is based on vaccination uniformly at random, i.e. all people have the same probability of receiving the vaccine. As stated in the article, this vaccination strategy is not optimal if the population structure is known exactly, but since this relevant population structure is generally hard to obtain for emerging diseases, vaccination uniformly at random might be the best feasible method.

Throughout we often use the superscripts "(hom)", "(net)", "(mult)", and "(house)", to refer to parameters and quantities associated with epidemics in respectively homogeneous mixing populations, network models, multi-type populations and populations consisting of households.

As a leading example we use the Markov SEIR epidemic model. In this model pairs of individuals make (close) contacts independently at a rate which might depend on the pair (depending on the population structure). If an infectious individual contacts a susceptible one, the susceptible one becomes latently infected (exposed) and stays so for an exponentially distributed time with mean $1/\delta$, after which the individual becomes infectious. An individual stays infectious for an exponentially distributed time with mean $1/\gamma$, after which he or she is removed, which might mean that the individual dies, he or she recovers with permanent immunity or is isolated in a 100% effective way. We also discuss the Markov SIR epidemic, in which there is no latent period (or $\delta = \infty$), but is the same as the Markov SEIR epidemic in all other respects. We assume that there are only a few initially infective individuals in the population and all others are susceptible.

1.2 Branching process results

In this section we need some notation: for $t > 0$, $\xi(t)$ is the random number of individuals infected by an infectious individual in the first t time units of his or her infectious period. Thus, $\xi(t)$ is a non-decreasing random process. Furthermore, define $\mu(t) = \mathbb{E}(\xi(t))$ as the expectation of $\xi(t)$. It is clear that $\mu(t)$ is also non-decreasing. For ease of exposition we assume that the derivative of $\mu(t)$ exists and is given by $\beta(t)$. Thus $\mu(t) = \int_0^t \beta(s)ds$. This assumption is not necessary and the results below can be generalized in a straightforward way to the case where $\mu(t)$ is not differentiable. From the theory of branching processes [1], we know that $R_0 = \mu(\infty) = \int_0^\infty \beta(s)ds$. In general there is no explicit expression for the Malthusian parameter α, only the implicit equation specifying α

$$1 = \int_0^\infty e^{-\alpha t}\beta(t)dt. \tag{1}$$

If $R_0 > 1$ (the situation we are interested in), this equation has exactly one real positive solution [1, p. 10], and serves as a definition of α.

If the length of the infectious period of an individual is distributed as the random variable I, and during his or her entire infectious period he or she infects other individuals at rate λ (that is, the infections form a homogeneous Poisson process with intensity λ), then $\beta(t) = \lambda\mathbb{P}(I > t)$. This gives that

$$R_0 = \int_0^\infty \beta(t)dt = \int_0^\infty \lambda\mathbb{P}(I > t)dt = \lambda\mathbb{E}(I). \tag{2}$$

Here we have used the standard equality $\int_0^\infty \mathbb{P}(X > t)dt = \mathbb{E}(X)$ for any non-negative random variable X (e.g. [5, Sec. 4.3]). From now on, for reasons of clarity, we assume that I has a density which is denoted by $f_I(t)$. We may relax these assumptions without further consequences. We deduce that

$$\begin{aligned} 1 = \int_0^\infty e^{-\alpha t}\beta(t)dt &= \int_0^\infty e^{-\alpha t}\lambda\mathbb{P}(I > t)dt = \lambda\int_{t=0}^\infty \int_{s=t}^\infty e^{-\alpha t} f_I(s)dsdt \\ &= \lambda\int_{s=0}^\infty \int_{t=0}^s e^{-\alpha t} f_I(s)dtds = \frac{\lambda}{\alpha}\int_0^\infty (1 - e^{-\alpha s})f_I(s)ds \\ &= \frac{\lambda}{\alpha}\mathbb{E}(1 - e^{-\alpha I}) = \frac{\lambda}{\alpha}(1 - \phi_I(\alpha)), \end{aligned} \tag{3}$$

where $\phi_I(\alpha) = \int_0^\infty e^{-\alpha t} f_I(t)dt = \mathbb{E}(e^{-\alpha I})$ is the Laplace transform of I or, which is the same, the moment-generating function of $-I$. Equation (3) gives an implicit equation for α.

If an infected individual only starts being infectious after a random latent period which is distributed as L and has density $f_L(t)$, and after this period he or she is infectious for another, independent, period which is distributed as I, during which he or she infects others at rate λ, then

$$\beta(t) = \lambda\int_0^t f_L(u)\mathbb{P}(I > t - u)du,$$

which is the convolution of $f_L(t)$ and $\beta_0(t)$, where $\beta_0(t)$ is the derivative of $\mathbb{E}(\xi(t))$ when the latent period is 0. This leads to

$$\begin{aligned} R_0 &= \int_{t=0}^\infty \lambda\int_{u=0}^t f_L(u)\mathbb{P}(I > t - u)dudt \\ &= \lambda\int_{u=0}^\infty \int_{t=u}^\infty f_L(u)\mathbb{P}(I > t - u)dtdu = \lambda\mathbb{E}(I), \end{aligned} \tag{4}$$

where we have used the same computations as in (2). We note that R_0 is independent of the latent period. Similarly we deduce that

$$1 = \int_{t=0}^{\infty} e^{-\alpha t}\lambda \int_{u=0}^{t} f_L(u)\mathbb{P}(I > t-u)dudt$$
$$= \lambda \int_{u=0}^{\infty}\int_{t=u}^{\infty} e^{-\alpha t} f_L(u)\mathbb{P}(I > t-u)dtdu$$
$$= \lambda \int_{u=0}^{\infty} e^{-\alpha u} f_L(u) \int_{t=0}^{\infty} e^{-\alpha t}\mathbb{P}(I > t)dtdu = \frac{\lambda}{\alpha}(1-\phi_I(\alpha))\phi_L(\alpha), \quad (5)$$

where ϕ_L is the Laplace transform of the random variable L. If L does not have a density the results above still hold. Note that if $L = 0$ with probability 1, then $\phi_L(\alpha) = 1$ and we obtain (3) again.

1.3 Homogeneously mixing populations

1.3.1 Constant infectivity

For SEIR epidemics in a (homogeneously) randomly mixing population, every time an individual makes a close contact, it is with a random other individual from the population, which is chosen uniformly at random, independently of other close contacts. During the emerging phase of an epidemic it is unlikely that an individual is chosen, who is no longer susceptible. Thus, we assume that all close contacts of infectious individuals are with susceptible ones. To make the above mathematically fully rigorous, we should consider a sequence of epidemics in populations of increasing size and derive limit results for this sequence of epidemics [2], but we leave out this level of technicality here.

If individuals each make close contacts independently at rate $\lambda^{(hom)}$, then we deduce from (4) and (5), that

$$R_0^{(hom)} = \lambda^{(hom)}\mathbb{E}(I) \qquad \text{and} \qquad 1 = \frac{\lambda^{(hom)}}{\alpha}(1-\phi_I(\alpha))\phi_L(\alpha).$$

In particular,

$$\frac{1}{R_0^{(hom)}} = \frac{(1-\phi_I(\alpha))\phi_L(\alpha)}{\alpha\mathbb{E}(I)}. \quad (6)$$

If I is exponentially distributed with mean $1/\gamma$ and there is no latent period, then $\phi_I(\alpha) = \frac{\gamma}{\gamma+\alpha}$ and $\phi_L(\alpha) = 1$, which leads to $R_0^{(hom)} = 1 + \alpha/\gamma$ as was deduced in the main text. If the latent period is exponentially distributed with mean $1/\delta$, then $\phi_L(\alpha) = \frac{\delta}{\delta+\alpha}$. Thus in the Markov SEIR model, (6) reads

$$\frac{1}{R_0^{(hom)}} = \frac{\gamma}{\gamma+\alpha}\frac{\delta}{\delta+\alpha},$$

whence

$$R_0^{(hom)} = \left(1+\frac{\alpha}{\gamma}\right)\left(1+\frac{\alpha}{\delta}\right)$$

1.3.2 Deterministic infectivity profile after latent period

We proceed by considering the (non-Markov) SEIR model in which, during the infectious period I being of random length, the close contact rate equals $h(\tau)$, where τ is the time since the infectious period starts. Note that we assume that $h(\tau)$ is non-random, i.e. identical for all infected individuals, but that the infectious period I may end after a random time hence being different for different individuals. We also allow for a random latency period L prior to the infectious period. In this case,

$$
\begin{aligned}
R_0^{(hom)} &= \int_{t=0}^{\infty}\int_{u=0}^{t} f_L(u)h(t-u)\mathbb{P}(I>t-u)dudt \\
&= \int_{u=0}^{\infty}\int_{t=u}^{\infty} f_L(u)h(t-u)\mathbb{P}(I>t-u)dtdu \\
&= \int_{u=0}^{\infty} f_L(u)\int_{t=0}^{\infty} h(t)\mathbb{P}(I>t)dtdu = \int_{0}^{\infty} h(t)\mathbb{P}(I>t)dt.
\end{aligned}
$$

Similarly, we obtain

$$
\begin{aligned}
1 &= \int_{t=0}^{\infty} e^{-\alpha t}\int_{u=0}^{t} f_L(u)h(t-u)\mathbb{P}(I>t-u)dudt \\
&= \int_{u=0}^{\infty}\int_{t=u}^{\infty} e^{-\alpha t} f_L(u)h(t-u)\mathbb{P}(I>t-u)dtdu \\
&= \int_{u=0}^{\infty} e^{-\alpha u} f_L(u)h(t)\int_{t=0}^{\infty} e^{-\alpha t}\mathbb{P}(I>t)dtdu \\
&= \phi_L(\alpha)\int_{0}^{\infty} e^{-\alpha t}h(t)\mathbb{P}(I>t)dt,
\end{aligned}
$$

whence,

$$
\frac{1}{R_0^{(hom)}} = \frac{\phi_L(\alpha)\int_0^\infty e^{-\alpha t}h(t)\mathbb{P}(I>t)dt}{\int_0^\infty h(t)\mathbb{P}(I>t)dt}. \tag{7}
$$

If $h(\tau)=\lambda$ is a constant then this equality can be rewritten as (6).

1.4 Configuration model network populations

1.4.1 The network

In this subsection we consider the configuration model network. In this network a fraction d_k of the n vertices (=individuals) has degree k, that is, a fraction d_k of the population has k other people it can have close contacts with, its acquaintances. The acquaintancies are represented by so-called bonds or edges. Out of all possible networks created in this way with given n and d_k's, we choose one uniformly at random. See [6, Ch.3], for more information on the construction of such networks.

We choose the (few) initial infective individuals all with equal probability (uniformly at random) from the population. If the population size n is large, then the probability that an initially infective individual has k acquaintances is d_k. However, by the construction of the network, the probability that an acquaintance of such an initially chosen infective has k acquaintances is not d_k; for $k=1,2,\cdots$ the probability is given by

$$
\tilde{d}_k = \frac{kd_k}{\sum_{j=0}^\infty jd_j} = \frac{kd_k}{\mu}, \quad \text{where } \mu = \sum_{j=0}^{\infty} jd_j,
$$

since an initial infective is k times as likely to be an acquaintance of an individual with degree k, than to be one of an individual with degree 1. Now, if an individual is infected during the early stage of an epidemic, then at least one of its acquaintances is no longer susceptible (i.e. its infector). However, if n is large, by the construction of the network the probability that its other acquaintances are still susceptible is close to 1. Hence, the expected number of susceptible acquaintances at the moment of infection of an individual infected during the early stages of the epidemic is

$$\sum_{k=1}^{\infty}(k-1)\tilde{d}_k = \sum_{k=1}^{\infty}(k-1)\frac{kd_k}{\mu} = \frac{\sum_{k=0}^{\infty}(k-\mu)^2 d_k}{\mu} + \mu - 1, \tag{8}$$

which is equal to κ as used in the main article.

1.4.2 The epidemic with constant infectivity

Consider an SEIR epidemic on the configuration network described above. Assume again that $f_L(t)$ is the density of the duration of the latent period and $f_I(t)$ the density of the duration of the infectious period. Assume that between every pair of acquaintances the rate of close contacts is $\lambda^{(net)}$ (i.e. close contacts occur according to independent Poisson processes with rate $\lambda^{(net)}$ per pair). The rate at which infection of a given acquaintance occurs at that time is $\lambda^{(net)}$ multiplied by the probability that the infector is infectious and has not previously infected this acquaintance, i.e.

$$\lambda^{(net)} \int_0^t f_L(s) e^{-\lambda^{(net)}(t-s)} \mathbb{P}(I > t-s) ds.$$

If the number of acquaintances of this infector is k, then the expected infectivity at time t is

$$(k-1)\lambda^{(net)} \int_0^t f_L(s) e^{-\lambda^{(net)}(t-s)} \mathbb{P}(I > t-s) ds.$$

Taking the mean over the number of acquaintances of an individual infected during the early stages of an epidemic, we obtain

$$\beta(t) = \kappa\lambda^{(net)} \int_0^t f_L(s) e^{-\lambda^{(net)}(t-s)} \mathbb{P}(I > t-s) ds.$$

This leads, after manipulations as performed in (2) and (3), to

$$\begin{aligned} R_0^{(net)} = \int_0^{\infty} \beta(t)dt &= \int_{t=0}^{\infty} \kappa\lambda^{(net)} \int_{u=0}^{t} f_L(u) e^{-\lambda^{(net)}(t-u)} \mathbb{P}(I > t-u) du dt \\ &= \kappa\lambda^{(net)} \int_{u=0}^{\infty} f_L(u) \int_{t=0}^{\infty} e^{-\lambda^{(net)}t} \mathbb{P}(I > t) dt du \\ &= \kappa\lambda^{(net)} \int_0^{\infty} e^{-\lambda^{(net)}t} \mathbb{P}(I > t) dt = \kappa(1 - \phi_I(\lambda^{(net)})) \end{aligned} \tag{9}$$

and

$$
\begin{aligned}
1 &= \int_0^\infty e^{-\alpha t}\beta(t)dt \\
&= \int_{t=0}^\infty e^{-\alpha t}\kappa\lambda^{(net)}\int_{u=0}^t f_L(u)e^{-\lambda^{(net)}(t-u)}\mathbb{P}(I > t-u)dudt \\
&= \kappa\lambda^{(net)}\int_{u=0}^\infty\int_{t=0}^\infty e^{-\alpha(t+u)}f_L(u)e^{-\lambda^{(net)}t}\mathbb{P}(I > t)dtdu \\
&= \kappa\lambda^{(net)}\phi_L(\alpha)\int_0^\infty e^{-(\alpha+\lambda^{(net)})t}\mathbb{P}(I > t)dt \\
&= \kappa\phi_L(\alpha)\frac{\lambda^{(net)}}{\alpha+\lambda^{(net)}}(1-\phi_I(\alpha+\lambda^{(net)})). \quad (10)
\end{aligned}
$$

Combining these observations gives

$$\frac{1}{R_0^{(net)}} = \phi_L(\alpha)\frac{\lambda^{(net)}}{\alpha+\lambda^{(net)}}\frac{1-\phi_I(\alpha+\lambda^{(net)})}{1-\phi_I(\lambda^{(net)})}. \quad (11)$$

If, as before, we consider the Markov SIR model in which $L = 0$ and I has an exponential distribution with mean $1/\gamma$, then (9) yields

$$R_0^{(net)} = \kappa((1-\phi_I(\lambda^{(net)})) = \kappa\frac{\lambda^{(net)}}{\lambda^{(net)}+\gamma} \quad (12)$$

and (10) yields

$$1 = \kappa\frac{\lambda^{(net)}}{\alpha+\lambda^{(net)}}(1-\phi_I(\lambda^{(net)}+\alpha)) = \kappa\frac{\lambda^{(net)}}{\lambda^{(net)}+\alpha+\gamma}.$$

The latter equality implies $\lambda^{(net)} = \frac{\gamma+\alpha}{\kappa-1}$, which inserted in the former gives

$$R_0^{(net)} = \frac{\gamma+\alpha}{\gamma+\alpha/\kappa}$$

as claimed in the main text.

If we consider the Markov SEIR epidemic in which the latent period has mean $1/\delta$ and the infectious period has mean $1/\gamma$, then $R_0^{(net)} = \kappa\frac{\lambda^{(net)}}{\lambda^{(net)}+\gamma}$ still holds, while (10) yields

$$1 = \frac{\delta}{\delta+\alpha}\frac{\kappa\lambda^{(net)}}{\lambda^{(net)}+\alpha+\gamma}, \quad (13)$$

which in turn implies

$$\lambda^{(net)} = \frac{(\gamma+\alpha)(\delta+\alpha)}{(\kappa-1)\delta-\alpha}.$$

Combining these observations gives that for the Markov SEIR epidemic

$$R_0^{(net)} = \frac{\gamma+\alpha}{\gamma\delta/(\delta+\alpha)+\alpha/\kappa}.$$

1.4.3 Deterministic infectivity profile after latent period

As in the homogeneous mixing case we now assume that the infectivity, conditional upon still being infectious, is a function of the time τ since the infectious period starts, say $\hat{h}(\tau)$ (later we assume that $\hat{h}$ is proportional to h as used in the homogeneous mixing population). Note that we assume that $\hat{h}(\tau)$ is not random, but that L and I are random and independent. In this case,

$$R_0^{(net)} = \kappa \int_{t=0}^{\infty} \int_{u=0}^{t} f_L(u)\hat{h}(t-u)e^{-\int_{s=0}^{t-u}\hat{h}(s)ds}\mathbb{P}(I > t-u)dudt$$
$$= \kappa \int_{u=0}^{\infty} f_L(u) \int_{t=0}^{\infty} \hat{h}(t)e^{-\int_{s=0}^{t}\hat{h}(s)ds}\mathbb{P}(I > t)dtdu$$
$$= \kappa \int_{t=0}^{\infty} \hat{h}(t)e^{-\int_0^t \hat{h}(s)ds}\mathbb{P}(I > t)dt.$$

Similarly, we obtain

$$1 = \kappa \int_{t=0}^{\infty} e^{-\alpha t} \int_{u=0}^{t} f_L(u)\hat{h}(t-u)e^{-\int_{s=0}^{t-u}\hat{h}(s)ds}\mathbb{P}(I > t-u)dudt$$
$$= \kappa \int_{u=0}^{\infty} e^{-\alpha u} f_L(u) \int_{t=0}^{\infty} e^{-\alpha t}\hat{h}(t)e^{-\int_{s=0}^{t}\hat{h}(s)ds}\mathbb{P}(I > t)dtdu$$
$$= \kappa\phi_L(\alpha) \int_{t=0}^{\infty} \hat{h}(t)e^{-\alpha t}e^{-\int_{s=0}^{t}\hat{h}(s)ds}\mathbb{P}(I > t)dt,$$

so,

$$\frac{1}{R_0^{(net)}} = \phi_L(\alpha)\frac{\int_{t=0}^{\infty}\hat{h}(t)e^{-(\alpha t+\int_{s=0}^{t}\hat{h}(s)ds)}\mathbb{P}(I > t)dt}{\int_{t=0}^{\infty}\hat{h}(t)e^{-\int_{s=0}^{t}\hat{h}(s)ds}\mathbb{P}(I > t)dt}.$$

1.4.4 Comparison of $R_0^{(hom)}$ and $R_0^{(net)}$

If we combine (6) and (11), and assume that α and the (constant) infection profiles (and thus ϕ_L and ϕ_L) are known and the same for both models, then

$$\frac{R_0^{(hom)}}{R_0^{(net)}} = \frac{\frac{1}{(\alpha+\lambda^{(net)})\mathbb{E}(I)}(1-\phi_I(\alpha+\lambda^{(net)}))}{\frac{1}{\alpha\mathbb{E}(I)}(1-\phi_I(\alpha))\frac{1}{\lambda^{(net)}\mathbb{E}(I)}(1-\phi_I(\lambda^{(net)}))}$$
$$= \frac{\mathbb{E}(I)\int_0^{\infty} e^{-(\alpha+\lambda^{(net)})t}\mathbb{P}(I > t)dt}{\left(\int_0^{\infty} e^{-\alpha t}\mathbb{P}(I > t)dt\right)\left(\int_0^{\infty} e^{-\lambda^{(net)}t}\mathbb{P}(I > t)dt\right)}.$$

To analyse this fraction, we introduce a random variable Y by its distribution function

$$\mathbb{P}(Y \leq y) = \frac{\int_0^y \mathbb{P}(I > t)dt}{\int_0^{\infty} \mathbb{P}(I > t)dt}, \quad \text{for } 0 \leq y < \infty.$$

Using this and recalling that $\mathbb{E}(I) = \int_0^{\infty} \mathbb{P}(I > t)dt$, we can write

$$\frac{R_0^{(hom)}}{R_0^{(net)}} = \frac{\mathbb{E}(e^{-\alpha Y}e^{-\lambda^{(net)}Y})}{\mathbb{E}(e^{-\alpha Y})\mathbb{E}(e^{-\lambda^{(net)}Y})}.$$

Since $\lambda^{(net)}, \alpha > 0$, we have that $e^{-\alpha x}$ and $e^{-\lambda^{(net)}x}$ are both non-increasing in x. Thus, by Chebyshev's integral inequality (or FKG inequality [5, p.86]), we have that $e^{-\alpha Y}$ and $e^{-\lambda^{(net)}Y}$ are positively correlated, whence $R_0^{(hom)} \geq R_0^{(net)}$.

The difference between $R_0^{(hom)}$ and $R_0^{(net)}$ is small if κ is relatively large compared to $R_0^{(hom)}$ and the standard deviation of the infectious period is not large compared to the mean. (See Figure 2 of the main article). It can easily be seen that the opposite makes the approximation worse. Infections taking place a long time after the start of an infector's infectious period contribute relatively little to α; on the other hand all infections make the same contribution to R_0. Also note, that if in the network model a given individual infects all of his/her acquaintances with large probability (say 99%) if he/she is infectious for a middle-long time (say T), then increasing the infectious period to $2T$ has little effect on the epidemic both on its size (which relates to R_0) and its speed (which relates to α). However, in a homogeneously mixing model, the offspring (which contributes to R_0) would double in expectation in this situation, while the speed of the epidemic would hardly change. Thus, if the standard deviation of the infectious period is large, we cannot ignore the large infectious periods which cause the discrepancy between $R_0^{(hom)}$ and $R_0^{(net)}$.

Now consider the second special case discussed above: the infectivity profile, conditional upon still being infectious, $\hat{h}(\tau)$ is not constant, but is proportional to $h(\tau)$ for the homogeneous mixing model, where τ is the time since an individual starts to be infectious. Let $\lambda := \hat{h}(\tau)/h(\tau)$. Then,

$$\frac{R_0^{(hom)}}{R_0^{(net)}} = \frac{\int_0^\infty h(t)\mathbb{P}(I>t)dt}{\int_0^\infty e^{-\alpha t}h(t)\mathbb{P}(I>t)dt}\,\frac{\int_{t=0}^\infty \lambda h(t)e^{-(\alpha t+\lambda\int_{\tau=0}^t h(\tau)d\tau)}\mathbb{P}(I>t)dt}{\int_{t=0}^\infty \lambda h(t)e^{-\lambda\int_{\tau=0}^t h(\tau)d\tau}\mathbb{P}(I>t)dt}.$$

As for the SEIR model with constant rates, we introduce a random variable Y' by its distribution function

$$\mathbb{P}(Y'\le y) = \frac{\int_0^y h(t)\mathbb{P}(I>t)dt}{\int_0^\infty h(t)\mathbb{P}(I>t)dt}, \quad \text{for } 0\le y<\infty.$$

Using this we can write

$$\frac{R_0^{(hom)}}{R_0^{(net)}} = \frac{\mathbb{E}(e^{-\alpha Y'}e^{-\lambda\int_0^{Y'}h(\tau)d\tau})}{\mathbb{E}(e^{-\alpha Y'})\mathbb{E}(e^{-\lambda\int_0^{Y'}h(\tau)d\tau})}. \tag{14}$$

Since λ and α are positive and $h(\tau)$ is a non-negative function, we have that $e^{-\alpha x}$ and $e^{-\lambda\int_{\tau=0}^x h(\tau)d\tau}$ are both non-increasing in x. Thus, copying the argument above, we have that $R_0^{(hom)} \ge R_0^{(net)}$. We note that although (14) does not explicitly depend on κ, the relationship between α and λ and $h(\tau)$ does and therefore the exact value of the right hand side does as well.

1.4.5 Example of a model where $R_0^{(hom)} < R_0^{(net)}$

The result $R_0^{(hom)} \ge R_0^{(net)}$ does not hold in general if $h(\tau)$ is a random function instead of a deterministic function, i.e. $h(\tau)$ is different for different people, following some distribution over stochastic processes. This is shown in the following extreme example.

We assume that every infective individual is infectious for exactly one point in time, at which he/she infects a random number of other individuals. In the homogeneous mixing case, with probability 1/3 an infectious individual infects on average 2 other individuals

at time 0 (relative to his/her time of infection), while with probability 2/3 he/she infects on average 1 other individual at time 1. This corresponds to

$$\mu(t) = 2\frac{1}{3} + \frac{2}{3}\mathbb{1}(t \geq 1),$$

leading to $R_0^{(hom)} = 4/3$ and $1 = 2/3 + (2/3)e^{-\alpha}$, which implies $e^{-\alpha} = 1/2$ (or $\alpha = \log[2]$).

In the corresponding network case we assume every individual has 3 acquaintances, so $\kappa = 2$. With probability 1/3 an infectious individual infects each of his/her susceptible acquaintances with probability $1 - e^{-2\lambda}$ independently at time 0, while with probability 2/3 he/she infects each of his/her susceptible acquaintances with probability $1 - e^{-\lambda}$ independently at time 1. Here λ is chosen such that $e^{-\alpha} = 1/2$.

For this model $\mu(t) = 2\left[(\frac{1}{3}(1-e^{-2\lambda}) + \frac{2}{3}(1-e^{-\lambda})\mathbb{1}(t>1)\right]$, leading to the equations

$$R_0^{(net)} = \frac{2}{3}(1-e^{-2\lambda}) + \frac{4}{3}(1-e^{-\lambda}) \qquad \text{and} \qquad 1 = \frac{2}{3}((1-e^{-2\lambda}) + (1-e^{-\lambda})).$$

Some algebra gives that $e^{-\lambda} = \frac{\sqrt{3}-1}{2}$, which implies

$$R_0^{(net)} = 2 - \frac{\sqrt{3}}{3} > \frac{4}{3} = R_0^{(hom)}.$$

1.5 Multi-type epidemics

For the SEIR epidemic in a multi-type population, we assume that there are ι types of individuals, labelled $1, 2, \cdots, \iota$ and again that the population is large. Additionally we assume that the number of individuals of each type is large, and in what follows we assume that there is no relevant depletion of susceptibles of any type during the initial stages of the epidemic. We assume that a fraction π_i of the community is of type i. Furthermore, we assume that not all close contacts lead to infection. However, we do assume that the probability that a close contact between a susceptible and an infectious individual leads to infection depends only on the time since infection of the infectious one, τ. This probability is random (i.e. different for different individuals) and is denoted by $\Lambda(\tau)$. Note that we assume that the distribution of $\Lambda(\tau)$ does not depend on the types of the individuals. The random function Λ incorporates the latent and recovered period, in the sense that before the end of the latent period and after recovery $\Lambda(\tau) = 0$. We use $g(\tau) = \mathbb{E}(\Lambda(\tau))$ for the expected probability of infection at age τ of a randomly selected individual. In an SIR epidemic the infectivity is often a function of τ conditioned on the individual still being infectious at time τ. In that case $g(\tau)$ can be written as $h(\tau)\mathbb{P}(I > \tau)$. Close contacts are not necessarily symmetric. That is, if individual x makes a close contact with individual y, then it is not necessarily the case that y makes a close contact with x. The rate of close contacts from a given type i individual to a given type j individual is λ_{ij}/n. Therefore the expected number of j-individuals that an infected i-individual infects up to its "age" (time since infection) t during the early stages of an outbreak when all individuals are susceptible is given by

$$m_{ij}(t) = \int_0^t a_{ij}(\tau)d\tau, \qquad \text{where} \qquad a_{ij}(\tau) = \lambda_{ij}\pi_j g(\tau). \tag{15}$$

The matrices $M(t)$ and $A(t)$ are defined by respectively $M(t) = (m_{ij}(t))$ and $A(\tau) = (a_{ij}(\tau))$. Furthermore, we define $M = M(\infty) = (m_{ij}(\infty))$ as the next generation matrix.

It is well-known that the basic reproduction number $R_0^{(mult)}$ is given by the dominant (i.e. "largest") eigenvalue of M, also denoted by ρ_M [3, 7].

To determine the epidemic growth rate, α, we use Equation (6.4) and the subsequent paragraphs from [7]. This translates into that the dominant eigenvalue of $\int_0^\infty e^{-\alpha\tau}A(\tau)d\tau$ should equal 1, where the integral is taken elementwise. Now we use that

$$\int_0^\infty e^{-\alpha\tau}a_{ij}(\tau)d\tau = \int_0^\infty e^{-\alpha\tau}\lambda_{ij}\pi_j g(\tau)d\tau = \lambda_{ij}\pi_j \int_0^\infty e^{-\alpha\tau}g(\tau)d\tau$$
$$= \frac{\int_0^\infty e^{-\alpha\tau}g(\tau)d\tau}{\int_0^\infty g(\tau)d\tau}m_{ij}(\infty).$$

Hence, ρ_A, the largest eigenvalue of the matrix $\int_0^\infty e^{-\alpha\tau}A(\tau)d\tau$ is given by ρ_M multiplied by $\int_0^\infty e^{-\alpha\tau}g(\tau)d\tau/(\int_0^\infty g(\tau)d\tau)$, where ρ_M is the largest eigenvalue of M. In particular this gives that

$$\frac{1}{R_0^{(mult)}} = \frac{\int_0^\infty e^{-\alpha\tau}g(\tau)d\tau}{\int_0^\infty g(\tau)d\tau}.$$

Notice that in the homogeneous case, i.e. the case with $\iota = 1$ and

$$\mu(dt) = g(t)\lambda_{11}dt,$$

we get the same relationship between α and $R_0^{(hom)}$ (as given in equation (7), with $h(\tau)\mathbb{P}(I > \tau) = g(\tau)\lambda_{11}$) as between α and $R_0^{(mult)}$, which implies that ignoring the population structure does not affect the estimates for R_0.

1.6 Household epidemics

Household epidemics are harder to study in this context (compared to homogeneous, network and multi-type epidemics) and already several papers are dedicated to these epidemics, e.g. [8]. In particular, there is no easy way to compute R_0 or α (instead other threshold parameters are often derived). Furthermore, if v_c is the critical vaccination coverage when vaccination is applied uniformly at random (i.e. the required control effort), then the relationship

$$v_c^{(house)} = 1 - 1/R_0^{(house)}$$

does not hold in general. Also, if the household structure is observed, then there are better vaccination strategies than vaccination uniformly at random [4]. (The same is true if the degrees of individuals are observed in the network model and if the types of individuals and their relative infectivities and susceptibilities are known in the multi-type model). However, in the article we consider the case where the population structure is hard to obtain. In that case vaccination uniformly at random seems to be the most natural vaccination strategy. Reproduction numbers for household epidemics and the relationships with vaccination uniformly at random and the epidemic growth rate are studied in great detail in [9] and some of the results will be repeated here.

For the household model we assume that the population is partitioned in n/m households (or groups or cliques) of equal size m. So, we assume that n is an integer multiple of the positive integer m. For a population where the households are not of equal size we refer to [10]. We consider only SEIR models in which individuals have constant infectivity during their infectious period. Individuals contact each other with global contacts at per-pair rate λ_G/n, while members of the same household make additionally local contacts

at per-pair rate λ_H. Note that, unlike in Section 1.5, we assume that close contact of an infective with a susceptible necessarily results in the infection of the latter.

We use the basic reproduction number $R_0^{(house)}$ as defined in [10, 9], since this is the parameter having interpretation closest to the common R_0 definition. This $R_0^{(house)}$ can be computed by considering one isolated household of size m, which has one initial infectious individual and $m-1$ susceptibles. Let $\mu_0 = 1$ and let μ_1 be the expected number of individuals in this household with whom the initial infective makes close contact during its infectious period (the first generation). Similarly μ_i is the expected number of individuals in the i-th generation, that is, the expected number of initially susceptible individuals which were not in the first $i-1$ generations, but have a close contact with a generation $(i-1)$ individual during its infectious period. Note that $\mu_i = 0$ for $i \geq n$. In [10] it is shown that $R_0^{(house)}$ is the unique positive x which solves

$$1 = \lambda_G \mathbb{E}(I) \sum_{i=0}^{m-1} \frac{\mu_i}{x^{i+1}}.$$

If the households are not all of the same size then the μ_i are replaced by household-size-biased averages, see Section 3.3. of [10].

In Section 2.6 of [9] it is shown that for SEIR epidemics R_0 estimates based on α and the homogeneous mixing assumption are conservative. We note that α is in general implicitly defined as the solution of an equation involving the infectivity profile of a household. Further arguments provided in [9] also show that in general

$$v_c^{(house)} \geq 1 - 1/R_0^{(house)}.$$

If we estimate v_c based on α and the homogeneous mixing assumption, then in most numerically analysed cases enough people are vaccinated. However, some counter examples are provided in [9].

In Figure 3 of the main text the dependence of R_0 and v_c on the relative contribution of the within household spread is illustrated for a household size distributions taken from Nigerian and Swedish datasets [11, 12].

2 Simulations

The simulations used in the article are performed in R and in MATLAB. In all simulations we use a Markov SEIR epidemic with the expected latent period twice the expected infectious period. This resembles the estimates for Ebola in West Africa [13], where the average time between infection and symptom onset and the start of the infectious period is estimated to be approximately 9.4 days (standard deviation 7.4 days) and the average time between symptom onset and hospitalization or death is approximately 5 days (standard deviation 4.7 days). Because the differences between the means of the infectious and latent periods and their corresponding standard deviations are relatively small, we use a Markov SEIR epidemic model in which both periods are exponentially distributed.

We simulated a Markov SEIR epidemic in a multi-type population 250 times in MATLAB. As a population we took the Dutch population in 1987 (approximately 14.6 million people) as used in [14], for which extensive data on contact structure are available. The

population is subdivided into six age groups (0-5, 6-12, 13-19, 20-39,40-59, 60+) and contact intensities are based on questionnaire data. For the simulations we use that the average infectious period $1/\gamma$ is 5 days, and the average latent period $1/\delta$ is 10 days. The infection rates λ_{ij} are chosen randomly for each simulation as follows. The data in Table 1 of [14] give estimates of m_{ij} $(i, j = 1, 2, \ldots, 6)$, where m_{ij} is the mean number of conversational partners per week in age class i of a typical individual in age class j. Using such conversations as a proxy for disease transmission, we assume that $\lambda_{ij} = cm_{ji}/\pi_j$, where π_j is the fraction of the Dutch population that are in age class j, estimated from Appendix Table 1 in [14], and c is a multiplicative constant chosen so that $R_0^{(mult)}$ has a specified value, which is sampled independently and uniformly from the interval between 1.5 and 3 for each simulation.

All simulated epidemics start with 1 infectious individual in each of the six age groups. We use two estimates of R_0. The first of these estimates is based on the average number of offspring from the people who were infected as 100th up to 1000th. We ignore the first 100 infecteds to ignore the effect of the initial stages of the epidemic, when the proportions of infecteds are still far from equilibrium. This procedure leads to a very good estimate of R_0 if the spread of the disease is observed completely. The second estimate is based on $\hat{\alpha}$, an estimate of the epidemic growth rate α, and neglects the multitype setting by assuming homogeneous mixing. We assume that we know γ and δ exactly and the estimate for R_0 is given by $(1 + \hat{\alpha}/\delta)(1 + \hat{\alpha}/\mu)$. The estimate $\hat{\alpha}$ is obtained from the development of the number of infectious people over time between the time the 100th individual becomes infectious and the time the 1000th individual becomes infectious, by using least square estimation of the natural logarithm of the number of infecteds against time. More specifically, if $t_{100}, t_{101}, \ldots, t_{1000}$ denote the times that these individuals become infected then $\hat{\alpha}$ is obtained by fitting a straight line to the points $(\log(i), t_i), i = 100, 101, \ldots, 1000$ using linear regression, so

$$\hat{\alpha} = \frac{901 \sum_{i=100}^{1000} \log(i) t_i - \sum_{i=100}^{1000} \log(i) \sum_{j=100}^{1000} t_j}{901 \sum_{i=100}^{1000} t_i^2 - \left(\sum_{i=100}^{1000} t_i\right)^2}.$$

In Figure 4(a) of the article we provide a scatter plot depicting the two estimates of R_0 for the 250 simulations. The ratio of the two estimates in the 250 simulations are summarized in Figure 4(b). In Figure S1 of this ESM the estimate or R_0 based on $\hat{\alpha}$ and the homogeneous mixing assumption is compared with the theoretical R_0, based on the full model. We see that the estimates are generally very good, as predicted by the theory.

To simulate epidemics on networks we use several networks from the Stanford Large Network Dataset collection [15]. In the main article we use a collaboration network in Condense Matter physics, because (i) this graph is undirected (if individual a can contact individual b, then b can contact a, (ii) this graph is large (23133 individuals) and (iii) the mean excess degree, κ is not extremely high. Individuals are acquaintances if they were co-authors of a manuscript posted on the e-print service arXiv in the condense matter physics section between January 1993 and April 2004. A manuscript with more than 2 authors leads to cliques (small groups in which everybody is acquainted to everyone else in the group). Since arguably many networks relevant for the spread of infectious diseases contain such cliques (households, workplaces and groups of friends), the presence of many cliques in collaboration networks is a desirable property.

Our simulations of Markov SEIR epidemics on all the networks considered are per-

formed in R, using the igraph package [16]. An epidemic starts with 10 uniformly chosen individuals which are at the start of their infectious period at time 0. We estimate the epidemic growth rate α based on the time between the total number of individuals which are infectious or recovered/deceased (the individuals that have shown symptoms) increases from 200 to 400. We exclude all simulations in which the total number of affected individuals stays below 400. The estimate of R_0 based on the real infection tree is obtained by looking at the epidemic from a generation perspective: All individuals infected by the initially infectious individuals are in generation 1, individuals infected by generation 1 infectives are in generation 2 etc. [10]. We consider as a reference generation the first generation in which there are 75 individuals (say generation k) and we divide the number of individuals in generation 2 up to $k+1$ by the number of individuals in generation 1 up to k. We exclude the initial individuals from the estimation of R_0, because those individuals are chosen uniformly at random and therefore independently of the population structure.

By trial and error investigation we tune the infection parameter λ such that the estimate of R_0 using the infection process is close to 2. Using this λ we run 1000 simulations. A typical graph of how the number of observed individuals (i.e. infectious + removed) is given in Figure S2(a). In part (*b*) we show the same graph but now we subtract 0.05 times the time to show that the growth of the number of individuals is indeed close to exponential over a large time.

Because of the mechanical way of estimating α, it is possible to have atypical epidemic trajectories, in which the estimation procedure is not good. Examples are (i) epidemics in which for example the exponential growth has not started yet at the time the 200th individual starts its infectious period or (ii) epidemics where just around the time the 200th or 400th of individual starts its infectious period a new part of the network is affected, where this new part contains many acquaintances within itself but is not well connected to the rest of the network. Such an event causes a sudden strong increase in the observed cases. These atypical trajectories are possible to identify if one observes the number of infectious individuals for a single epidemic and better estimates can be obtained in this way. We deal with this problem by not considering the simulations which give the 5% lowest and 5% highest estimates for α.

In Figure S3 we provide a scatter plot of the two estimates of R_0 for the simulations used, we see that in the vast majority of the simulations, the estimate of R_0 based on the estimated α and the homogeneously mixing assumption is conservative. We note that the two estimates are hardly correlated.

We further summarize our data in Figure 5 of the article, and in Figure S4. In which the ratio and difference of the R_0 estimate based on the epidemic growth rate and assuming homogeneous mixing, and the R_0 estimate based on the observed infection process, are given.

We also analyse the spread of SEIR epidemics on 2 other networks described in the Stanford Large Network Dataset collection [15]. The first is the collaboration network in Astro Physics, which is obtained in a similar way as the collaboration network in Condense Matter Physics. This network is slightly smaller than the Condense Matter Physics network and has a higher κ (approximately 64 instead of 21). The analysis is performed similarly to the analysis of the Condense Matter Physics collaboration network. Boxplots of the estimates of R_0 using the real infection process, the estimates of R_0 using the epidemic growth rate and assuming homogeneous mixing, as well as a boxplot of the ratio of those estimates, are given in Figure S5.

We see that the two estimates are close, but that the simpler estimate assuming homogeneous mixing is slightly conservative for all three empirical networks, which is consistent with the theoretical result for the configuration model.

The second alternative network is a part of the facebook social network from [15]. This part is relatively small and we restrict ourselves to the largest connected component (containing 1034 individuals). This network has a high mean degree (51.7) and mean excess degree (93.5). Because of its relatively small size, and the observation that some substantial parts of the network are connected to the other parts of the network through only a few connections, the estimate of R_0 through the epidemic growth rate is less good. We also have to adapt the bounds for estimating R_0 from the infection tree (as a reference generation the first generation in which there are 40 individuals), and we estimate the epidemic growth rate based on the time between the total number of individuals which are infectious or recovered/deceased increases from 150 to 350. Furthermore, in order to obtain quicker convergence the 7 initial infectious individuals are chosen proportional to their number of acquaintances, which gives individuals with many acquaintances a higher probability of being initially infectious. Boxplots of the estimates of R_0 using the real infection process, the estimates assuming homogeneous mixing and using the epidemic growth rate, as well as a boxplot of the ratio of those estimates, are given in Figure S5.

References

[1] Jagers P. Branching Processes with Biological Applications. New York: Wiley; 1975.

[2] Ball F, Donnelly P. Strong approximations for epidemic models. Stochastic Process Appl. 1995;55(1):1–21. Available from: http://dx.doi.org/10.1016/0304-4149(94)00034-Q.

[3] Diekmann O, Heesterbeek H, Britton T. Mathematical Tools for Understanding Infectious Disease Dynamics. Princeton University Press; 2013.

[4] Ball FG, Lyne OD. Optimal vaccination policies for stochastic epidemics among a population of households. Math Biosci. 2002;177:333–354.

[5] Grimmett G, Stirzaker D. Probability and random processes. 2nd ed. Oxford university press; 1992.

[6] Durrett R. Random graph dynamics. Cambridge University Press; 2006.

[7] Diekmann O, Gyllenberg M, Metz JAJ, Thieme HR. On the formulation and analysis of general deterministic structured population models. I. Linear theory. J Math Biol. 1998;36(4):349–388. Available from: http://dx.doi.org/10.1007/s002850050104.

[8] Ball F, Mollison D, Scalia-Tomba G. Epidemics with two levels of mixing. Ann Appl Probab. 1997;7:46–89.

[9] Ball F, Pellis L, Trapman P. Reproduction numbers for epidemic models with households and other social structures II: comparisons and implications for vaccination. Math Biosci. 2016;274:108–139.

[10] Pellis L, Ball F, Trapman P. Reproduction numbers for epidemic models with households and other social structures. I. Definition and calculation of R_0. Math Biosci. 2012;235(1):85–97.

[11] Demographic, Nigeria. Health survey (NDHS). Problems in accessing health care NDHS/National Population Commission. 2003;p. 140.

[12] Statistics Sweden. Statistical Yearbook of Sweden 2014. Statistics Sweden; 2014.

[13] WHO Ebola Response Team. Ebola virus disease in West Africa – the first 9 months of the epidemic and forward projections. N Engl J Med. 2014;371:1481–1495.

[14] Wallinga J, Teunis P, Kretzschmar M. Using data on social contacts to estimate age-specific transmission parameters for respiratory-spread infectious agents. Am J Epidemiol. 2006;164(10):936–944.

[15] Leskovec J, Krevl A. SNAP Datasets: Stanford Large Network Dataset Collection; 2014. http://snap.stanford.edu/data.

[16] Csardi G, Nepusz T. The igraph software package for complex network research. InterJournal. 2006;Complex Systems:1695. Available from: http://igraph.org.

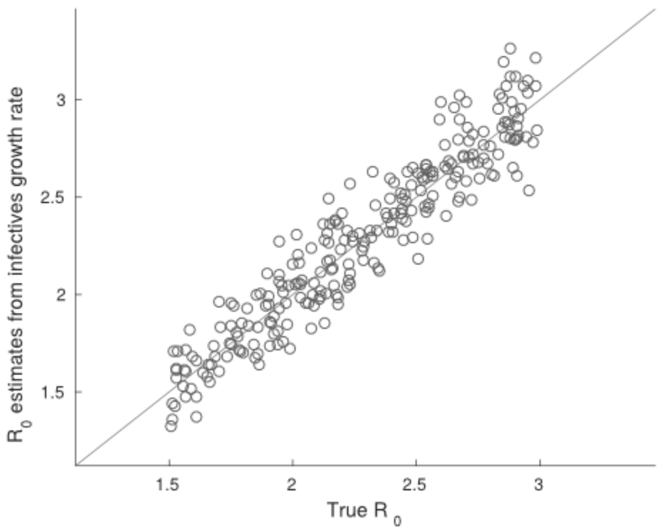

Figure S1: The estimated basic reproduction number, R_0, for a Markov SEIR model in a multi-type population as described in [14], based on the homogeneous mixing assumption and the estimated epidemic growth rate, α, against the computed R_0 based on the full model. The infectivity is chosen at random, such that the theoretical R_0 is uniform between 1.5 and 3. The estimate of α is based on the times when individuals become infectious.

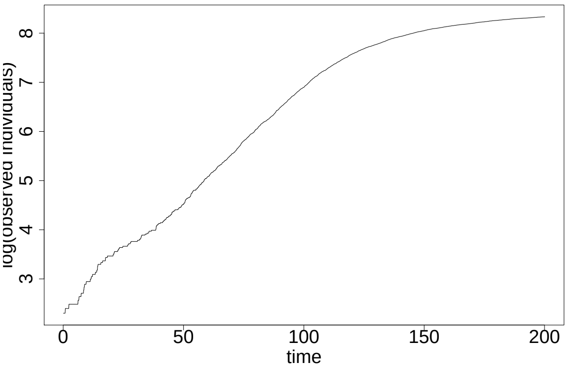

(a)

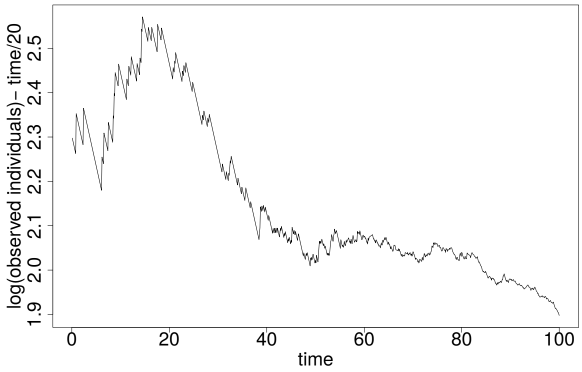

(b)

Figure S2: (a) A typical graph of the log of the number of observed (infectious + removed) individuals as a function time. (b) The same function minus 0.05 times the time.

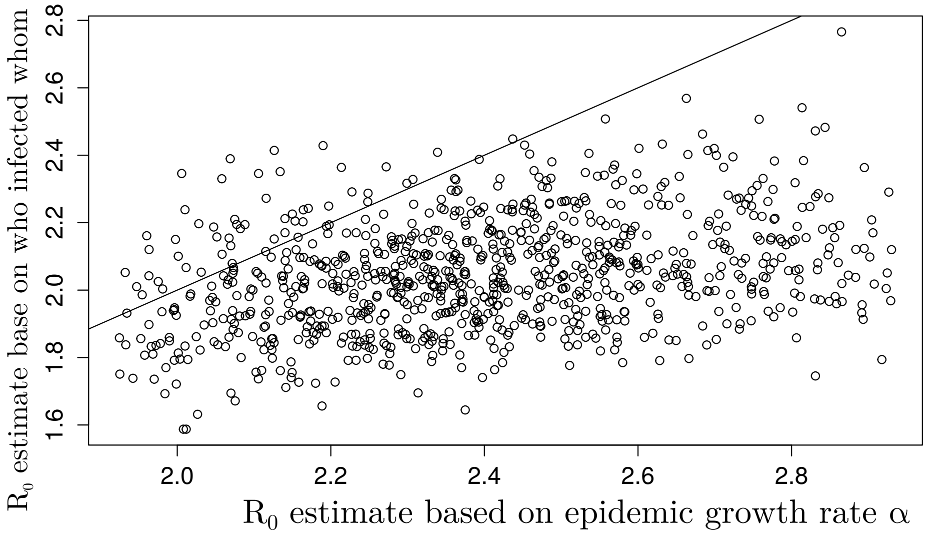

Figure S3: Scatter plot of estimates of R_0 assuming homogeneous mixing and using the estimated epidemic growth rate, and estimates based on the real infection process (who infected whom) in the collaboration network in Condense Matter Physics. 1000 simulations are used and the simulations with the 50 lowest and 50 highest estimated epidemic growth rates are not represented in the scatter plot. The line shows where the two estimates are equal.

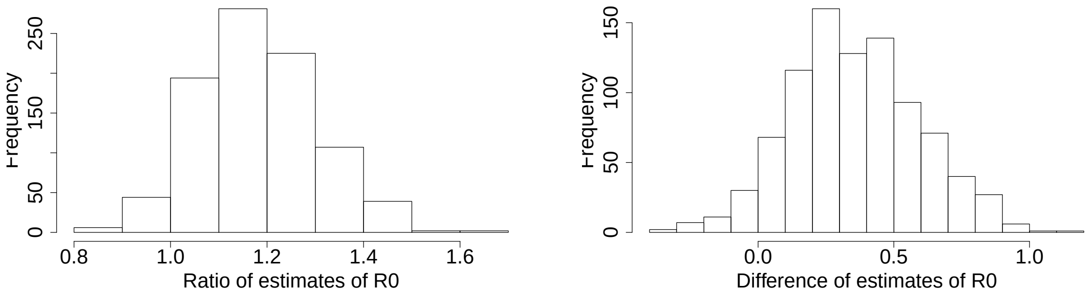

(a) (b)

Figure S4: Histograms of the ratio (a) of and difference (b) between the estimates of R_0 assuming homogeneous mixing and using the estimated epidemic growth rate, and estimates based on the real infection process in the collaboration network in Condense Matter Physics. 1000 simulations are used and the simulations with the 50 lowest and 50 highest estimated epidemic growth rates are not represented in the histograms.

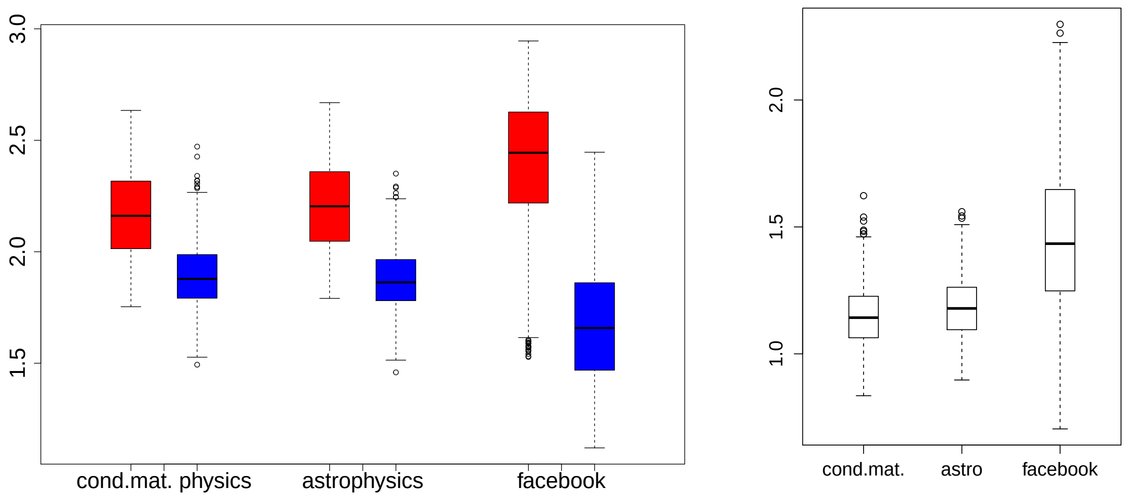

(a) (b)

Figure S5: Boxplots of estimates of R_0 for three networks from [15]: The condensed matter physics and astrophysics collaboration network and a facebook social network graph. In (a) the estimates assuming homogeneous mixing and using the epidemic growth rate are plotted in red, while the estimates based on the real infection process are plotted in blue. In (b) the ratios of the two estimates of R_0 for each simulation are summarized.
